# Oxidative Stress and Inflammation: From Mechanisms to Therapeutic Approaches

**DOI:** 10.3390/biomedicines10040753

**Published:** 2022-03-23

**Authors:** Juan Gambini, Kristine Stromsnes

**Affiliations:** Freshage Research Group, Department of Physiology, Faculty of Medicine, Insitute of Health Research-INCLIVA, University of Valencia and CIBERFES, Avda. Blasco Ibañez, 15, 46010 Valencia, Spain; kristine.stromsnes@uv.es

Oxidative stress and inflammation are two phenomena that are directly involved in practically all pathologies and especially in aging. Oxidative stress, which is associated with the redox state, constitutes an important mechanism in many physiological processes, such as adaptations to physical exercise, cell signaling, and hypothalamic appetite regulation. Inflammatory mediators are known to be essential in mechanisms such as the generation of gastric mucus for the protection of the stomach and for the repair of tissues via the mobilization of stem cells. However, when these two phenomena are deregulated, their actions are harmful. In this Special Issue, we ask ourselves several questions: How and when should we allow or block oxidative stress and inflammation? What is the advisable dose of antioxidant or anti-inflammatory therapy associated with aging? Are diet, physical exercise, and decreased psychological stress the best therapies for oxidative stress and inflammation control? In this Special Issue, we have published 14 articles: 7 original research articles and 7 reviews. They describe everything from the inflammatory and oxidative processes involved in different diseases to functional foods containing molecules that counteract these phenomena.

In order to reflect upon the comments made in this Special Issue in a coherent way, the publications have been divided into two sections that we will comment on below.

Environmental pollution has become a major public health risk in recent decades, causing millions of deaths worldwide. Among pollutants, we find harmful or harmful components that can be both physical, chemical, and biological. Identifying these compounds is the starting point to combat them.

In their original research article, Heba Al Housseiny et al. [1] have identified and reflected how the exposure of a product called PM2.5, which is derived from the combustion of matter, is associated with a decrease in lung function, impaired immunity, and exacerbations in lung disease. Their results show how these particles are capable of reducing cell viability and increasing the inflammatory parameters and oxidative stress in lung epithelial cells. These data suggest that effective mitigation strategies are necessary to address not only the identification of polluting particles but also their possible neutralization and treatments that avoid their toxicity [1].

Acute respiratory distress syndrome is one of the pathologies of the respiratory system. This condition presents with the acute onset of respiratory failure that leads to bilateral pulmonary infiltrates seen on chest radiographs, hypoxemia, etc., and that can result in direct lung lesions, pneumonia, sepsis, and even death. Globally, it has an incidence of around 15 to 80/100,000 inhabitants per year, and so far, no beneficial drug therapy has been developed; however, it has been found that platelets play a fundamental role in this disease. Therefore, Chuan-Mu Chen et al. [2] describe studies that show the possible benefits of antiplatelet therapy for prevention and treatment via the control of platelet activity and describe the mechanisms of action that are involved.

Dry eye syndrome affects approximately 40% of the adult population. It manifests as hyperemia, glare, fatigue, and eye irritation. The most critical consequences of this desiccant stress are inflammation and damage to the ocular surface, which, in severe cases, can lead to blindness, scarring, opacification or ulceration of the cornea, and blurred vision. Treatments for this condition include lubricating drops, anti-inflammatory therapy, or immunosuppressants such as cyclosporine. However, all of these treatments can cause adverse effects that limit their use. For this reason, in their original article, Dmitry V. Chistyakov et al. [3] have tested a therapy with a new ophthalmic formulation containing a zileuton solution based on dimethylsulfoxide. Their results showed the high efficacy of the proposed therapy. On the one hand, DMSO decreased the activity of pro-inflammatory protagladins, and on the other hand, zileuton decreased the cytokine levels released by T cells. In this way, the two compounds presented synergistic activity that opens the door to more effective anti-inflammatory therapies against said pathology [3].

Gender differences in cardiovascular diseases (CVD) as well as the identification of biomarkers for disease detection are challenges that are of great importance today. In the original study by Laura Bordoni et al. [4], they recruited 547 individuals comprising both men and women with or without cardiovascular disease to identify useful biomarkers to characterize gender differences in CVD. The study evaluated blood parameters such as plasma membrane fluidity, erythrocyte lipid hydroperoxides, and trimethylamine (TMA). Lower plasmatic TMA was observed in male CVD patients compared to in the healthy male controls, while higher levels of TMA were measured in female CVD patients with respect to the female controls. Diphenyl−1-pyrenylphosphine (DPPP) was significantly lower in male CVD patients compared to in the healthy controls, while no significant changes were measured in females with or without CVD. The results obtained allowed Laura Bordoni et al. to conclude that TMA could be an effective marker for the detection and prevention of CVD and that the DPPP value could be predictive biomarker of CVD in men [4].

Breast cancer remains as one of the most common cancers today. As advances in diagnostic and treatment methods continue to become more effective, the survival rate continues to increase. However, the chemotherapy that is administered, although effective, still presents important adverse reactions, such as difficulty concentrating and remembering or alterations in processing speed. Patients may also experience symptoms of gastrointestinal (GI) issues, such as diarrhea and vomiting as well as long-term hepatotoxicity or liver damage. In their review, Taurean Brown et al. [5] describe how the series of symptoms experienced by cancer patients are interconnected and mediated by inflammatory responses. Therefore, new therapeutic approaches could help improve the quality of life in breast cancer patients after treatment [5].

Protecting the kidneys is essential in the management of diabetes-derived problems. There is good evidence to illustrate the causal link between oxidative stress and the progression of diabetes-related kidney disease (DKD). Currently, the only therapeutic options that are for DKD are limited to systemic interventions for the metabolic changes related to diabetes, such as dyslipidemia, hypertension, and hyperglycemia. Given the important role that oxidative stress plays in various aspects of kidney injury, the redox imbalance could be considered to be potential therapeutic target for renal failure in diabetic patients. This review article written by Keiichiro Matoba et al. [6] shows recent therapeutic approaches to prevent DKD by targeting the antioxidant effects of newly developed antihyperglycemic agents.

In their review, Spyridon Methenitis et al. [7] discuss the possibility of exercise-induced muscle damage being a suitable model of inflammation for the evaluation of nutraceutical anti-inflammatory properties. Low-grade, subclinical inflammation is one of the main pathophysiological mechanisms underlying most chronic and non-communicable diseases. In this sense, the use of functional foods, those with bioactive compounds and, in this case, with an anti-inflammatory capacity, could be a model to take into consideration in humans. The capacity of these compounds could be highly valued since it is known that bioavailability is a key point in their effectiveness [7].

Compounds with pharmacological activities with the possibility of having great therapeutic power have been found in nature.

Telomere shortening and oxidative stress have been linked to aging and patients with cancer and inflammatory diseases. In 2016, the *New England Journal of Medicine* published an article showing how danazol, a synthetic androgen, promotes telomere lengthening in circulating human leukocytes, comparing its effects to a hydroethanolic root extract that is known to stimulate telomerase activity. These findings open the door for treatments for associated diseases, telomeres, or aging. In this Special Issue, Isabelle Guinobert et al. [8] show how danazol, similar to extracts from the Astragalus mongholicus plant, has positive effects on telomerase-dependent telomere lengthening in human lymphocytes in their review. These findings create possibilities for new treatments for diseases that are associated with aging [8].

Diospirin is a medicinal compound derived from the Diospyros lotus, which has anticancer, antituberculous, and antileishmanial activities against Leishmania donovani. Adnan Shahidullah et al. [9] describe a modulatory role in the inflammatory response induced by lipopolysaccharides (LPS) in mouse macrophages through the inhibition of nitric oxide and several cytokines via the ER-stressed calcium-p38 MAPK/CHOP/Fas pathway. These effects have not been shown previously and could have important therapeutic uses [9].

Prolonged strenuous exercise can induce unfavorable biological changes and symptoms, including inflammatory responses that can lead to intestinal barrier dysfunction. Many athletes use non-steroidal anti-inflammatory drugs to treat inflammation-induced algesthesia. Sihui Ma et al. [10] have verified how hyperimmunized milk supplementation, obtained after immunizing cows against specific antigens, protects intestinal function, exerts anti-inflammatory effects, and promotes the development of immunity against various pathogens. This could be a viable nutritional approach for preventing intense exercise-induced symptoms.

Caloric restriction has been shown to be a powerful intervention to extend both lifespan and health span in various animal models, from yeast to primates. Furthermore, in humans, caloric restriction has been found to induce cardiometabolic adaptations that are associated with better health. Daniele La Russa et al. [11] studied the long-term effects of caloric restrictions on the inflammatory and redox balance in aged, obese rats. They found that caloric restriction not only induces weight loss but also positively modulates both the anti-inflammatory and antioxidant pathways while also improving the circulating adiponectin levels in obese old rats. Along with achieving normal weight, these adaptations, which are induced by caloric restriction, suggest that the redox and inflammatory imbalance in obese aged rats appears to be caused by obesity rather than by aging [11].

Microglia play an important role in the development of neurodegenerative diseases, but the mechanisms of action have yet to be specified. When activated, they can express pro-inflammatory cytokines that act on the surrounding brain and spinal cord, something that can have a detrimental effect on nerve cells when they acquire a chronic inflammatory function and promote neuropathologies. In their bibliographic review, Tamotsu Tsukahara et al. [12] set out to clarify the mechanism of action by which the porcine liver decomposition product, which is rich in phospholipids, exerts beneficial effects on cognitive functions in healthy humans. The authors propose that this food is function due to its possibility of enhancing visual memory and delay recall as well as to improve Hasegawa’s Dementia Scale-Revised scores and the Wechsler Memory Scale in healthy adults and discuss whether it would be convenient to use it in patients with neurodegenerative diseases. Additionally, they reinforce their hypothesis by outlining other recent findings showing the bidirectional interactions between lysophospholipids, microglia, and age-related neurodegenerative diseases [12].

Fatty acids (n-3 PUFA) are long chain polyunsaturated fatty acids that contain 18, 20 or 22 carbon atoms and that exert a multitude of benefits on human health. Specifically, eicosapentaenoic acid (EPA) and docosahexaenoic acid (DHA) have been found to produce both cardioprotective and responses by modulating membrane phospholipids, thereby improving cardiac mitochondrial functions and energy production. Dietary supplementation of n-3 PUFAs has been found to reduce the endothelial cell apoptosis and mitochondrial dysfunction associated with oxidative stress. Additionally, it has the capacity to restore myocardial performance and vascular reactivity by counteracting the release of pro-inflammatory cytokines in vascular tissues and in the myocardium. In their review, Francesca Oppedisano et al. [13] have summarized the molecular mechanisms underlying the antioxidant and anti-inflammatory effects of n-3 PUFAs on vascular and cardiac tissues and their implication in the prevention and treatment of cardiovascular diseases.

Due to medical advances and lifestyle changes, the life expectancy of the population has increased. For this reason, it is important to achieve healthy aging by reducing the risk factors that cause age-related damage and pathologies. Through nutrition, one of the pillars of health, we are able to modify these factors by modulating the intestinal microbiota. In their bibliographic review, Elisa Sanchez-Morate et al. [14] show that diets such as the Mediterranean and Oriental diets exert healthy effects, mainly due to the high consumption of polyphenols and fibers, which interact with the intestinal bacteria, thereby generating beneficial effects on the body. Additionally, the low consumption of fats in these diets favors the state of the microbiota, thereby further contributing to the maintenance of good health [14].

In conclusion, this Special Issue addresses the roles that oxidative stress and inflammation play in different pathologies, such as ophthalmological, respiratory, and cardiovascular diseases as well as in obesity, cancer, and diabetes. In addition, without forgetting that pharmacological treatments are essential for their treatment, it has been revealed how natural products, diets, or certain foods could positively influence action against these diseases.
